# Recent Science

**Published:** 1895-05-18

**Authors:** 


### Recent Science.

It is a satisfactory sign of the times that such a publication as *Science Progress* should achieve success. When so much in scientific journalism is being sacrificed to the desire to be readable, and while writers are made to feel that, at all hazards, the deepest facts must be made comprehensible to the shallowest understandings, it is a pleasure to find a periodical which appeals to an educated audience, and, although devoting itself to the most abstruse sciences and the most recent investigations, is, to such an audience, eminently readable and full of interest.

When electricity and all its ways were vague subjects of speculation, even philosophers were not expected to know all the secrets of its operations; but since it has been tamed, brought into common service, and sold in the open market, it may surprise some to find how far we are from knowing all about it. An extremely interesting article by Professor Lodge on "Light and Electrification" points out a series of facts which have hitherto received but little attention, and adds to them by observations of his own regarding the effect of light in facilitating electrical discharge. Roughly, one may say that an electroscope charged with electricity, and standing in a non-conducting atmosphere, will retain its charge for a considerable period, the rate at which its leaves fall indicating its "leak." Now it is found that the rate of "leak" is much increased by the apparatus being exposed to rays of ultra-violet light, and with this curious peculiarity, that a negative charge is lost much more quickly than a positive one, and in fact that a freshly-cleaned metal surface, exposed to light, actually acquires a slight positive charge. But it is now shown that while with clean bright metal surfaces the difference between the rates of discharge is so enormous that it is practically true to say that one pours away while the other is retained, still the leakage of positive also is increased by illumination.

With dirty surfaces, however, and with certain metals positive electricity escapes much more readily. The important observation was also made that the efficacy of polarised light in promoting leakage varied according to the plane of its polarisation in regard to its angle of incidence. The question then arose whether the effect was due to some quality in the light or something in the nature of the surface, and by experiment it was shown that the latter was the case; for, whereas light which had been used once, and had then been reflected on to another surface, still retained its efficacy, such a metal as palladium appeared to get rapidly "fatigued" by the action of light in regard to its capacity for showing leakage under its influence. The deductions to be gathered from these observations are promised in a future number.

Dr. Buckmaster contributes an article on "The Anti-toxin of Diphtheria," in which he well describes the present position of our knowledge on the subject. A careful perusal of this article will serve to damp the dogmatism of many who speak as if we had already fathomed the mystery. The facts which have been observed are many, and their bearing on each other are most interesting, but it is clear that of knowledge we have as yet but little, and that we still await the master mind which shall demonstrate the meaning of those experimental results which at present are but being utilised in an empirical fashion. Those who imagine that anti-toxins are specific, each for their own toxins, may well take note of this statement: "The serum of animals immune to rabies or titanus is powerfully anti-toxic to cobra venom. From these observations the products of cell activity which accumulate in the blood of animals immune to tetanus, diphtheria, or cobra poison, are all of an anti toxic character similar in nature, and the view that anti-toxic serum is specific for a definite bacillus or its products must be modified."

There are in *Science Progress* for this month several other articles of great interest, of which that by Dr. Waller on the relation between trophic action, nerve cells, and the direction taken by functional impulses is of special importance to physiologists and physicians.

